# Influences of *Bacillus subtilis* and *fructooligosaccharide* on growth performances, immune responses, and disease resistance of Nile tilapia, *Oreochromis niloticus*

**DOI:** 10.3389/fvets.2022.1094681

**Published:** 2023-01-12

**Authors:** Arporn Panase, Mongkol Thirabunyanon, Jongkon Promya, Chanagun Chitmanat

**Affiliations:** ^1^Program in Biotechnology, Faculty of Science, Maejo University, Chiang Mai, Thailand; ^2^Faculty of Fisheries Technology and Aquatic Resources, Maejo University, Chiang Mai, Thailand

**Keywords:** probiotics, prebiotics, feed additives, immunomodulation, non-specific immunity, immune gene expression

## Abstract

The present study investigated the effects of *Bacillus subtilis* and fructooligosaccharide (FOS) on growth performances, immunity improvement, and disease resistance of Nile tilapia (*Oreochromis niloticus*). The fish (24.5 ± 1.6 g) were fed a basal diet (G1), diets supplemented with 1 g/kg (G2), 3 g/kg (G3) and 5 g/kg (G4) of FOS as well as diets supplemented with 1 × 10^9^ CFU/g (G5), 3 × 10^9^ CFU/g (G6) and 5 × 10^9^ CFU/g (G7) of *B. subtilis* for 56 days. After the feeding trial, the complement C3, *IL-1*β, *TNF-*α, IFN-γ, *hsp70* gene expression in the liver was then analyzed by a quantitative Real-time PCR. Then, fish were infected with *Streptococcus agalactiae*, and the survival rate was recorded. The results showed that FOS and *B. subtilis* had no significant effect (*P* > 0.05) on growth performances and survival rate. Lysozyme activity was significantly greater in the G4, G5, G6, and G7 groups. Also, all fish fed FOS and *B. subtilis* showed significantly (*P* < 0.05) higher respiratory burst activity than other groups. The expressions of complement *C3, IL-1*β, *TNF-*α, *IFN-*γ, and *hsp-70* in the liver were significantly higher for fish fed 5 g/kg of FOS as well as for fish that received any concentration level of *B. subtilis* (*P* < 0.05) used in the study. After the *S. agalactiae* challenge test, the survival rate of fish-fed diets supplemented with FOS and *B. subtilis* was slightly higher than for the control group. The results indicated that FOS an*d B. subtilis* could stimulate immune responses and immune-related genes in tilapia. However, further investigation of other prebiotics or herbs in combination with *B. subtilis* is encouraged at molecular levels and screening for beneficial metabolites that may increasingly improve digestive enzymes, growth performances, and health benefits in tilapia. In addition, on-farm experiments are needed.

## 1. Introduction

Probiotics and prebiotics are promising feed additives for a sustainable aquaculture. Nile tilapia (*Oreochromis niloticus*) is a predominant freshwater cultured fish due to its fast growth rate, suitability for aquaculture, and high marketability. It is now farmed in over 100 countries worldwide (1, 2). However, the rapid expansion of Nile tilapia farming has been negatively disturbed by infectious diseases and climate uncertainties, causing huge economic losses. The most common pathogenic bacteria affecting Nile tilapia are *Streptococcus agalactiae, Flavobacterium columnare*, and *Aeromonas hydrophila* (3). For the prevention and treatment of these diseases, chemicals and antibiotics have been widely used. However, overuse of these substances has led to the development of resistant bacteria, residue in the flesh, and destruction of the microbial population in aquatic environments (4, 5). In recent decades, sustainable strategies have been developed for using antibiotics *via* natural bioactive compounds, which have been widely applied in aquaculture (6–8). Among them, prebiotics and probiotics are of tremendous potential because they are safe for customers and the environment as well as are able to stimulate beneficial bacteria in the gastrointestinal tract of host fish (9, 10).

Prebiotics are non-digestible food ingredients that can certainly influence a host fish by enhancing growth performance and interacting with bacteria in the gastrointestinal tract; this, in turn, improves the host's health (11). The most common prebiotics used in aquaculture includes mannanoligosaccharide (MOS), fructooligosaccharide (FOS), inulin, and galactooligosaccharide (12). FOS, short and medium chains of β-D-fructans, can be fermented by certain bacteria such as lactobacilli and bifidobacterial so after dietary supplemented, it would improve the growth and survival of such bacteria in the GI tract of animals (13). FOS supplemented feed could enhance intestinal enzymes activities, absorptive ability, and histological features of intestinal villi and subsequently improve the feed utilization and growth performance of Nile tilapia (14). Tilapia fed a diet supplemented with 20–30 g FOS/ kg (3%) enhanced immune responses, reduced oxidative stress, and increased survival rates when infected with *A. hydrophila* (15) while Abd El-Gawad et al. (16) reported that 2% dietary FOS was the most suitable and beneficial dose for Nile tilapia. Positive effects of FOS on fish growth and immune responses were reported in other aquatic animals; for example, Caspian roach (*Rutilus rutilus*) fed 2 and 3% FOS improved digestive enzyme activity, enhanced growth performance, and significantly elevated resistance to a salinity stress challenge (17). Tambaqui (*Colossoma macropomum)*, fed only 0.1 and 0.5% FOS presented a better growth performance (18). Dietary supplementation of FOS at a dose of 1% increased growth performances and stimulated the immune responses of juvenile stellate sturgeon (*Acipenser stellatus*) (19).

Besides prebiotics associated with the aquafeed additive business, Probiotics are referred to living microorganisms that provide the host benefit by improving the intestinal microbial balance, inhibiting the growth of pathogenic microorganisms, increasing feed nutrient utilization, and stimulating the immune responses. Microorganisms have been broadly applied as probiotics in aquaculture including *Lactobacillus, Bacillus*, and *Saccharomyces* species. *Bacillus* spp. are widely used in aquafeeds for feed utilization improvement, growth performance promotion, innate immune regulation, disease resistance, and water quality improvement for a sustainable aquaculture (20). These non-pathogenic bacteria are able to produce robust spores so they can endure high temperatures, dehydration, and resistance to gastric environments (21). The optimal concentration of *B. licheniformis* in juvenile tilapia diets was ≥ 4.4 × 10^6^ CFU/g of their feed. With this amount in their diets fish exhibited enhanced growth performance, immune response, and disease resistance. In addition, the supplementation of *B. subtilis* in the food given to red sea bream at 1 × 10^8^ and 1 × 10^10^ CFU/kg of their diet was shown to increase the growth, feed utilization, health condition and immune response of the fish (22). There were several studies on the use of *Bacillus subtilis* in Nile tilapia with different dosages and various results. Tilapia fed diets supplemented with *Bacillus* and lactic acid bacteria had significantly better growth performances than a control feed (23). Dietary supplementation with *Bacillus* sp. KUQ1 and *Bacillus* sp. KUQ2 increased lysozyme, phagocytic, and respiratory burst activity in tilapia (3). A feed supplement of *B. subtilis* C-3102 at low dose (10^5^ CFU/g) induced upregulation of intestinal cytokine expression (IL-1b, TGF-β and TNF-α) and downregulation of intestinal *hsp70* (24). Tilapia received *B. subtilis* additive feed at a concentration of 5 × 10^6^ CFU/g improved the innate immune system (lysozyme and phagocytic activities of macrophages) and reduced the stress under a high stocking density (25). A dietary 0.3% *B. subtilis* was the effective prophylactic against *Streptococcus agalactiae* infection (26). The application of the *B. subtilis, Saccharomyces cerevisiae* and *Aspergillus oryzae* mixture had no significant effect on the growth performances of Nile tilapia, while the cumulative mortality after *A. hydrophila* and *S. iniae* challenge decreased (27).

Although numerous studies showing the benefits of probiotics and prebiotics on fish growth improvement, immunity stimulation, and pathogenic bacteria resistance enhancement, to confirm the results of *Bacillus* spp. and FOS feed additives in tilapia cultivation; therefore, the effects of dietary supplementation of commercial *Bacillus subtilis* and FOS on growth performances, expression of immune-related genes, non-specific immunity responses, and resistance against *Streptococcus agalactiae* infection in tilapia were investigated.

## 2. Materials and methods

### 2.1. Fructooligosaccharide and *Bacillus subtilis* preparations

Quantum Hi-Tech Biological Co., Ltd., China, supplied the fructooligosaccharide (FOS) used in this study. Its appearance was a white or light-yellow powder without any visible impurities. The product's composition was 1-kestose (1-kestotriose; GF2), nystose (GF3), 1F-fructofuranosylnystose (GF4), and other components, including bacterial, molds, and yeast, which were not more than 10 CFU/g. The commercially available probiotic product used (Greentech Aquaculture co., LTD., Thailand) contained 1 × 10 ^9^ CFU/g *Bacillus subtilis*.

### 2.2. Diet preparation

The basal diet (HiGrade 9951, CPF Thailand) was commercially available and contained 30% crude protein, 3% lipid, and 2 % crude fiber which was sufficient to support the optimal growth of Nile tilapia. This basal feed, with no supplementation (prebiotics or probiotics), was used as a control (G1) diet. The basal diet was supplemented with three levels of FOS; 1 (G2), 3 (G3), and 5 (G4) g/kg or three levels of *B. subtilis* 1 × 10^9^ (G5) 3 × 10^9^ (G6), and 5 × 10^9^ CFU/g (G7). These concentrations of FOS and *B. subtilis* were sprayed onto 1 kg of the basal diet. These diets were coated with 20 mL of fish oil and air-dried at room temperature for 24 h, then stored in sealed plastic bags at 4°C for further use.

### 2.3. Fish and feeding design

Healthy reverted male Nile tilapia (average body weight 24.5 ± 1.6 g) were obtained from a local fish farm and acclimated for 2 weeks in (2 m × 2 m) cages. The fish were fed to satiation with a commercial diet twice daily at 08.00 a.m. and 4.00 p.m. After acclimation, 420 fish were randomly divided into seven groups and stocked in 2m × 2m cages in triplicate at a rate of 20 fish per cage. The experiment was conducted for 56 days. Fish were fed twice daily at a rate of 5% of the body weight, and the fish were weighed every 2 weeks to adjust the feed amount.

### 2.4. Growth performance and survival measurement

At the end of the feeding trial, fish were not fed for 24 h, then anesthetized using 2-Phenoxyethanol (300 mg/L) (99%, MERCK, USA) before sample collection. Fish in each cage were weighed for growth performances, and the survival rate was recorded. The growth parameters were calculated according to the following formula: Weight gain (WG, %) = (final weight–initial weight) × 100; Average daily gain (ADG) = 100 × (final body weight – initial body weight) / experimental period; Feed Conversion Ratio (FCR) = quantity of feed offered/weight gain; Survival (%) = (final number of fish/initial number of fish) × 100.

### 2.5. Fish sample collection

Three fish from each cage (9 fish per treatment group) were randomly selected for blood collection at the termination of the feeding trial (56 days). First, fish were anesthetized using 2-Phenoxyethanol (300 mg/L), and cleaned using alcohol, with special care taken around the anus to avoid contamination. Then, 1 mL of blood was taken from the caudal vein using a plastic syringe. For each fish tested, 0.5 mL of blood was placed into heparin tubes to determine respiratory burst activity. Another 0.5 mL of blood was transferred into Eppendorf tubes without anticoagulation and allowed to clot at room temperature for 4 h. The serum was then separated, moved into new tubes, and stored at −20°C so that lysozyme activity could be measured.

### 2.6. Immunological assays

#### 2.6.1. Lysozyme activity

With slight modifications, lysozyme activity was measured following Parry, Chandan and Shahani (17). Briefly, 25 μL of fish serum was loaded into a 96-well plate in triplicate. Then, 175 μL of *Micrococcus lysodeikticus* suspension [0.2 mg mL^−1^ in sodium phosphate buffer (pH 6.2)] was added to each well. The reaction was determined through a spectrophotometer at 540 nm, and the absorbance level was recorded every 1 min for 10 min. The lysozyme activity in fish serum was calculated as a reduction in A540 of 0.001 min^−1^ and expressed as mL^−1^units.

#### 2.6.2. Respiratory burst activity

Superoxide anion (O^2−^) was used to determine respiratory burst activity through nitroblue tetrazolium (NBT) reduction reactions, which were performed by modifying the protocol of Secombes (28). Briefly, white blood cells (6 × 10^6^ cells) were added to 96-well plates in triplicate batches. Then, 25 μL of NBT was added to each well, and they were incubated at room temperature for 2 h. After incubation, the supernatant was discarded, and 150 μL of 100% methanol was added to each one to fix the cells. The wells were then washed with a 70% methanol solution twice. Finally, a 150 μL amount of potassium hydroxide (2 M KOH) and 100 μL amount of dimethyl sulfoxide (DMSO) were added to each well. The mixture was thoroughly mixed, and the reaction was measured at an absorbance level of 655 nm (A655) *via* a spectrophotometer.

### 2.7. Gene expression

#### 2.7.1. RNA extraction and cDNA synthesis

Liver tissues were collected from three fish per treatment group for total RNA extractions. An amount of 20 ng μL^−1^ for the liver was used. According to the manufacturer's protocols, total RNA was extracted using a PureLink RNA Mini Kit (Ambion, USA). The quality of the RNA was measured spectrophotometrically (NanoDrop 2000, Thermo scientific) and with gel electrophoresis (1% agarose gel). Total RNA was converted to cDNA Complementary DNA (cDNA) using a SensiFAST™ SYBR^®^ No-ROX Kit (Bioline, UK) following the manufacturer's protocols.

#### 2.7.2. qRT-PCR analysis

The primer sequences of C3, *IL-1*β, *TNF-*α, *IFN-*γ, *hsp70* genes, as well as the β-actin housekeeping genes, are shown in Table 1. The quantitative qPCR (PCRmax Eco 48 Real-time qPCR System, PCRmax, UK) was used for gene expression. First, the SYBR green method was applied to determine gene expression *via* RT-PCR (SensiFast SYBR Lo-Rox kit, Bioline). The amplification conditions were as follows: 45 cycles (95°C for 10 s, 63°C for 30 s, and 72°C for 30 s). Afterward, the relative expression levels of target genes were analyzed using the 2^−ΔΔCT^ method (29).

**Table 1:** Primers used for detection of a target gene.

**Gene**	**FWD or REV**	**Sequence (5^′^-3^′^)**	**Product size (bp)**	**References**
*Actin*	Forward	TGT GAG TCT ACA GTG AGG AGC	95	(62)
	Reverse	CCC AGA TCT AAA GCC ATT CTG C		
C3	Forward	TGG CAA TGA GAG GTT CCG	196	
	Reverse	TGC TGT TGT AGG TGG TTT CG		
*IL-1β*	Forward	TGCTGAGCACAGAATTCCAG	60	(63)
	Reverse	GCTGTGGAGAAGAACCAAGC		
*TNF-α*	Forward	GAGGTCGGCGTGCCAAGA	119	(64)
	Reverse	TGGTTTCCGTCCACAGCGT		
*IFN-γ*	Forward	TGACCACATCGTTCAGAGCA	128	
	Reverse	GGCGACCTTTAGCCTTTGT		
*hsp70*	Forward	TGGAGTCCTACGCCTTCAACA	238	
	Reverse	CAGGTAGCACCAGTGGGCAT		

### 2.8. Challenge test

*S. agalactiae* was freshly prepared by inoculating a single colony of the bacteria into Nutrient Broth (NB, Himedia) and culturing it at 32°C for 24 h. It was harvested through centrifugation at 5,000 rpm at 4°C for 10 min, followed by washing and then resuspension in 0.85% NaCl solution. The *S. agalactiae* suspension was adjusted to 10^8^ CFU/ml with 0.85% NaCl before injection. At the end of the feeding trial, 10 fish were randomly collected from each group and intraperitoneally injected with 0.1 ml of *S. agalactiae* (10^8^ CFU/ml) and the mortality rate was recorded for 14 days.

### 2.9. Statistical analysis

Results are expressed as the mean values ± standard deviation (SD). Differences among treatments were determined using a one-way analysis of variance (ANOVA) with the statistical software SPSS Version 15.0. A *post-hoc*, Duncan test was applied to examine significant differences between treatments. Significant differences were accepted at *P* < 0.05.

### 2.10. Ethical approval

The experiments were conducted according to the norms established by the Maejo University Animal Care and Use Committee (MUACUC, Approval Number MACUC025F/2565).

## 3. Results

### 3.1. Growth performances and survival rates

**Figure 1:**
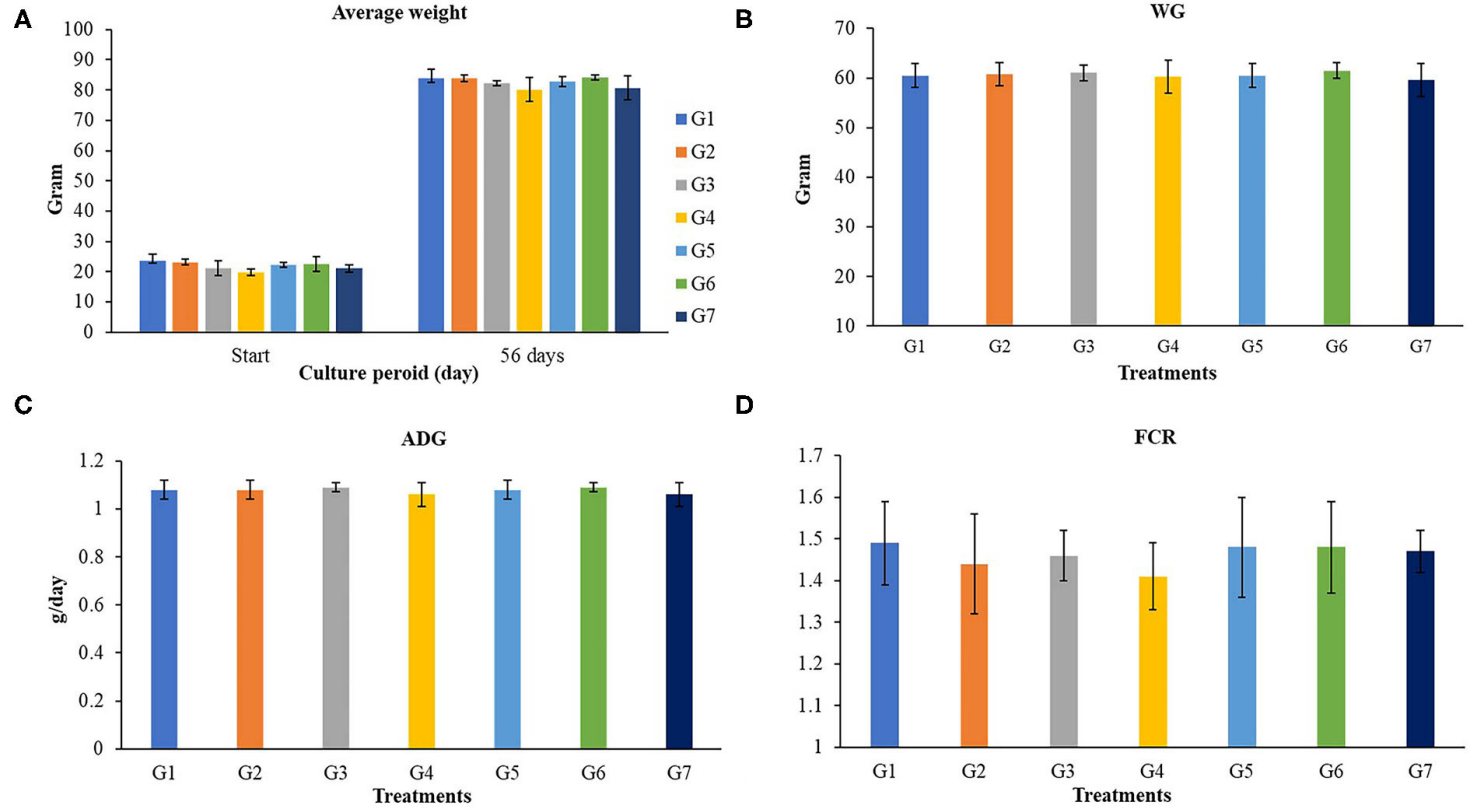
Growth performances of Nile tilapia fed a control feed and diets supplemented with different concentrations of FOS and *B. subtilis* for 56 days. WG, Weigh gain; ADG, average daily growth; FCR, Feed conversion rate. **(A)** (Average Weight), **(B)** (WG), **(C)** (ADG), and **(D)** (FCR).

**Figure 2:**
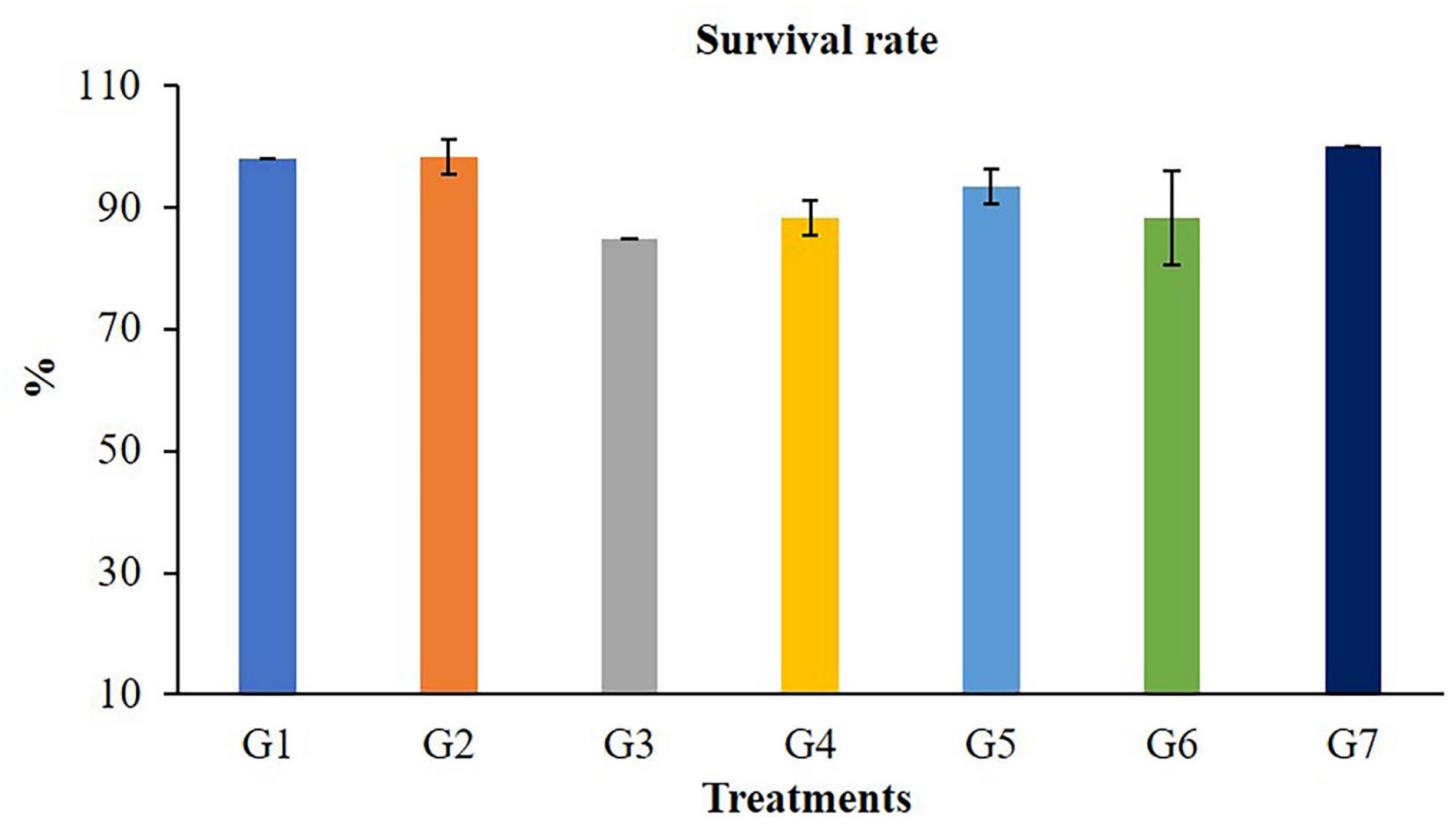
Survival rate of Nile tilapia after fed a control feed and diets supplemented with different concentrations of FOS and *B. subtilis* for 56 days.

The growth performances of Nile tilapia after a 56-day feeding trial with FOS and *B. subtilis* are presented in Figure 1. The average weight (Figure 1A), weight gain, WG (Figure 1B), average daily gain, ADG (Figure 1C), feed conversion ratio, FCR (Figure 1D), and survival rates (Figure 2) were not significantly different from the control group (*P* > 0.05). Neither the supplementation of FOS (1–5 g/kg feed) or *B. subtilis* (1–5 × 10^9^ CFU/g) did not promote growth performances and survival rates in this study.

### 3.2. Immune parameters

**Figure 3:**
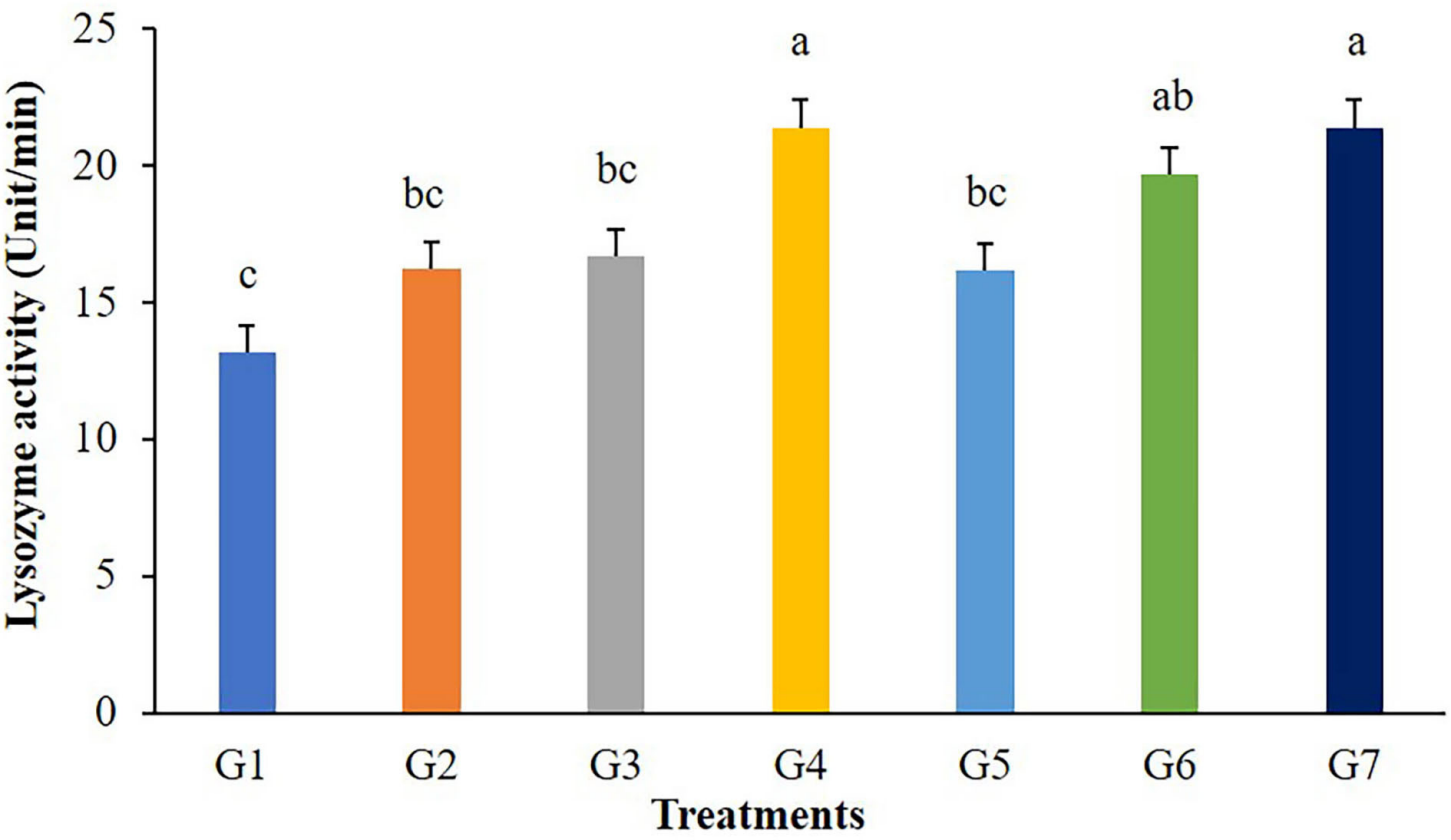
Lysozyme activity of Nile tilapia fed with FOS and *B. subtilis* for 56 days (*n* = 5). Bars with different letters indicate significant difference (*P* < 0.05).

**Figure 4:**
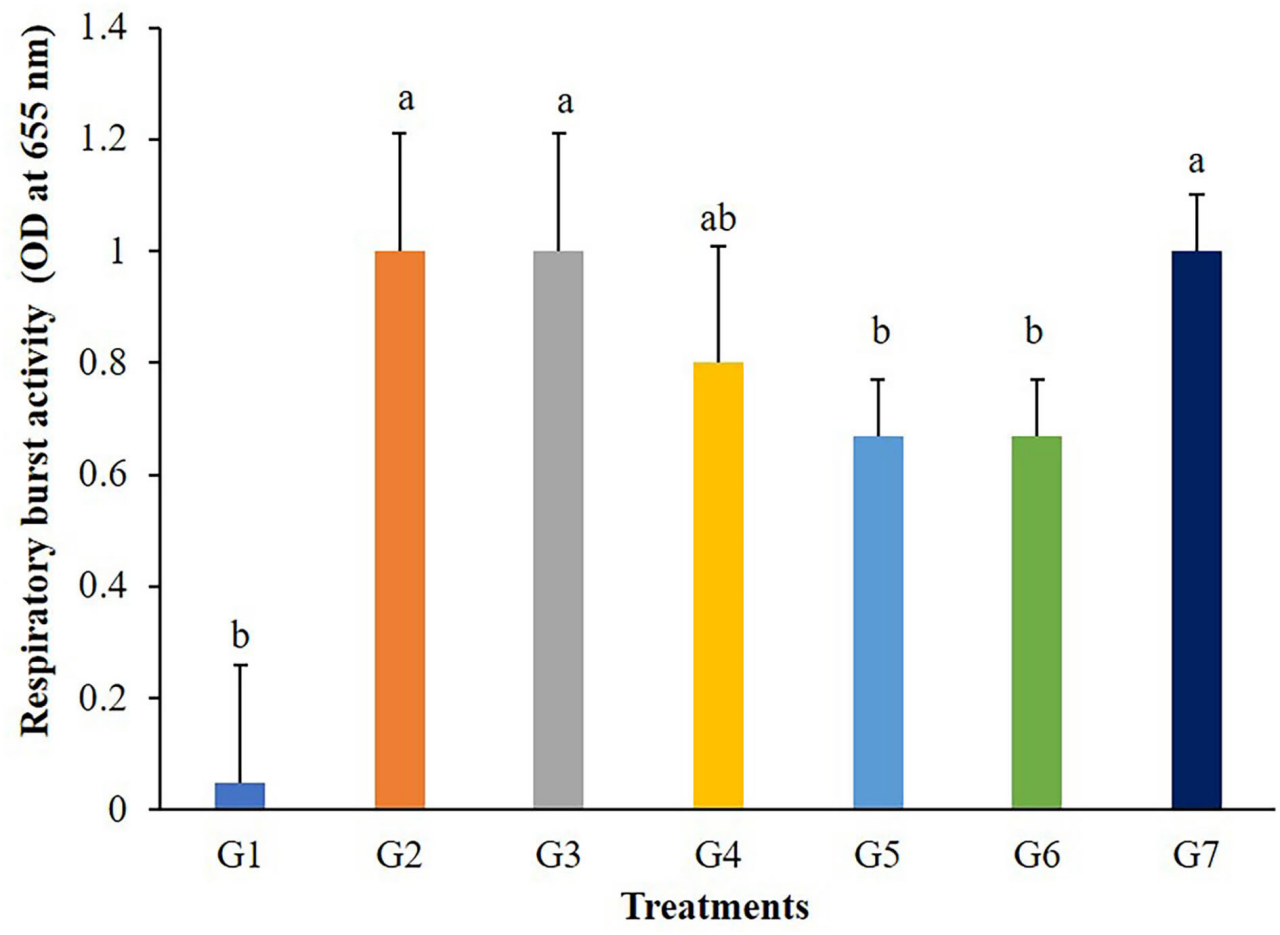
Respiratory burst activity of Nile tilapia fed with FOS and *B. subtilis* for 56 days (*n* = 5). Bars with different letters indicate significant difference (*P* < 0.05).

The highest values of lysozyme were found in fish fed G4 and G7 diets (Figure 3). Lysozymes might be enhanced in fish fed with 5 g/kg of FOS (G4) or 5 × 10^9^ CFU/g *B. subtilis* (G7) but those in G2, G3, and G5 treatments were not different from the control. In addition, significant differences (*P* < 0.05) in respiratory burst activity were observed in G2, G3, and G7 after 56 days of the feeding trial (Figure 4).

### 3.3. Gene expression in the liver of Nile tilapia

**Figure 5:**
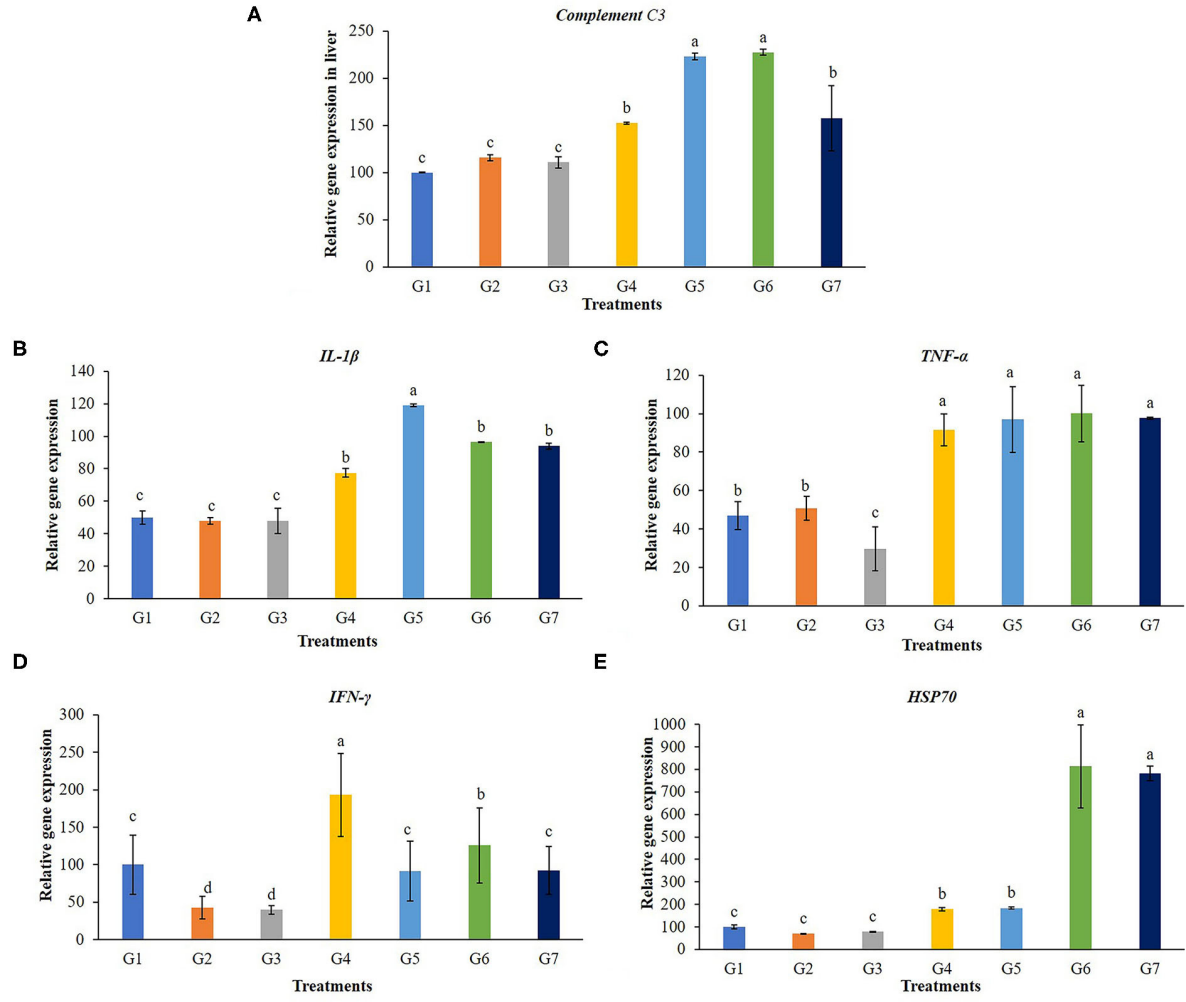
Gene expression in the liver of complement C3, interleukin 1beta (*IL-1*β), tumor necrosis factor (*TNF-*α), interferon gamma (*IFN-*γ) and heat shock protein 70 (*hsp70*) of Nile tilapia fed with FOS and *B. subtilis* for 56 days. **(A)** Complement C3, **(B)** Beta (*IL-1*β), **(C)** tumor necrosis factor (TNF-α), **(D)** interferon gamma (*IFN-*γ), and **(E)** heat shock protein 70 (*hsp70*).

A transcript of the immune-related gene expression tests for the liver of the tilapia is given in Figure 5. Complementary C3 and *IL-1*β were significantly up-regulated in the liver of tilapia fed with 5 g FOS/kg feed (G4) and those fed with *Bacillus* additive diets (G5, G6, and G7) (*P* < 0.05). The *TNF-*α gene expression levels in fish fed with 5 g FOS/kg feed (G4) and all *B. subtilis* treatment groups were significantly higher compared with the control group and other treatment groups (G2 and G3) (*P* < 0.05). Furthermore, higher *TFN-*γ gene expression was found in the fish fed with 5 g FOS/kg feed (G4) over the control group and other treatment groups (*P* < 0.05). In addition, a higher level of *hsp70* gene expression was found in the 5 g FOS/kg feed (G4) and all concentrations of *Bacillus* additive feeds (G5, G6, and G7) over those of the control group and other groups (*P* < 0.05).

### 3.4. Challenge test

**Figure 6:**
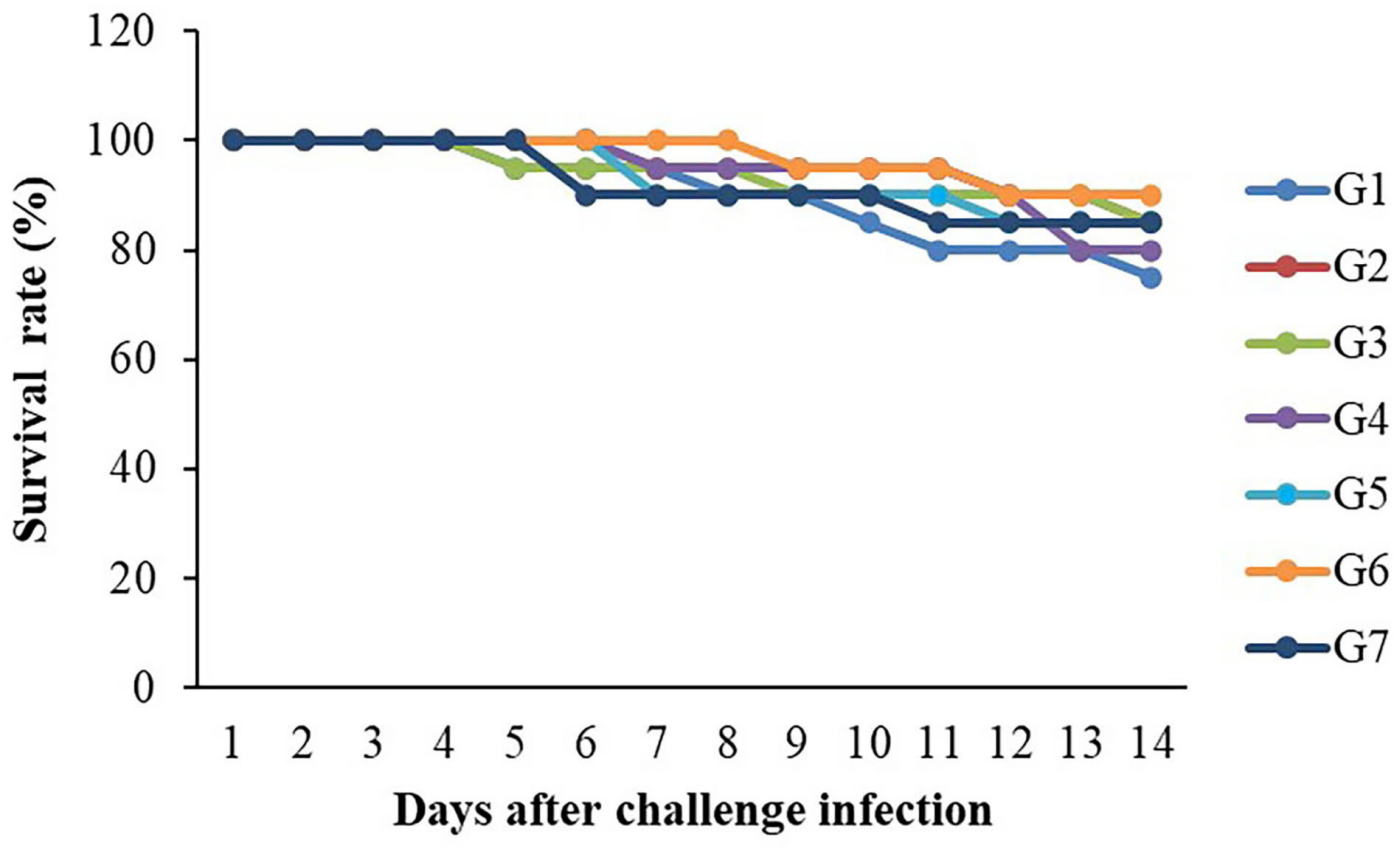
Survival rates (%) of Nile tilapia fed with FOS and *B. subtilis* after challenge with *S. agalactiae* 1 × 10^8^ CFU/ml (*n* = 20) for 14 days.

The 14-day challenge test indicated that the highest survival rate was found in the G6 group, whereas the lowest survival rate was observed in the control group (Figure 6). However, there were no significant differences in survival rates (*P* > 0.05) between the control and the supplemented diet groups. Clinical signs of infected fish included abnormal swimming, darkened color and less of an appetite. In addition, hemorrhages on the surfaces of their bodies and on their livers were found to be larger than those found in normal fish.

## 4. Discussion

Prebiotic and probiotic feed additive applications have been considered as promising alternative approaches for preventing diseases in fish and shellfish aquaculture. They provided better feed utilization, promoted growth performances, improved survival rate, boosted immunological responses, and enhanced animal welfare (30–33). *B. subtilis* supplementations resulted in superior growth performances, as has been reported in Dabry's sturgeon, *Acipenser dabryanus*; hybrid Hulong grouper, *Epinephelus fuscoguttatus* × *E. lanceolatus*; and tongue sole, *Cynoglossus semilaevis* (34). Probiotics possibly regulate the various autochthonous bacteria in a gastrointestinal tract that help to improve digestion or increase appetite of host organisms thus leading to be better nutrient absorption and improved growth. There are several studies that have reported the improvement of growth performances in Tilapia after *B. subtilis* feeding of in Nile tilapia. For example, Nile tilapia fed a basal diet supplemented with *B. subtilis* MRS11 at 1 × 10^8^ CFU/g of feed for 60 days improved growth performances, intestinal morphology, immunity, and the survival rate after challenge with *Streptococcus iniae* (35). The dietary supplementation of mixed *Bacillus* strains (Sanolife^®^ PRO-F) to Nile tilapia, *O. niloticus* at 0.5–1 g/kg diet improved the growth, feed utilization, antioxidant property and immune parameters (36). A dietary supplement of *B. subtilis* HAINUP40 can effectively improve the growth performance, immune responses, and disease resistance of Nile tilapia (37). However, the present study revealed no significant improvement in growth and feed utilization. Similarly, the application of *Bacillus* sp. KUAQ1 and *Bacillus* sp. KUAQ2 in tilapia fry produced no effect (*P* > 0.05) on average weight, average daily growth, specific growth rate or feed conversion ratio after an 8-week feeding trial (3). The possible reasons for this difference may be due to the difference in probiotic activities, beneficial bacteria interactions in the fishes' guts, the amount of the probiotic products added, strain/species composition, its viability, as well as types of feeds, feeding durations, and experimental conditions.

Prebiotics can increase feed utilization efficiency by promoting growth of gut microbiota in fish leading to lower feed conversion and increase growth rates. Unfortunately, the supplementation of FOS (1–5 g/kg feed) did not promote growth performances and survival rates in the present study. These results were in agreement with previous investigations reported, where juvenile large yellow croaker was used, *Larimichthys crocea* (0.2–0.4% FOS) (38) and Atlantic salmon (*Salmo salar*) (1% FOS) However, the results of this study did contrast with studies on Caspian roach (*Rutilus rutilus*) fry (1–3% FOS), tambaqui (*Colossoma macropomum)* (0.1 and 0.5% FOS), stellate sturgeon (*Acipenser stellatus*) juveniles (1% FOS), and blunt snout bream (*Megalobrama amblycephala*) (0.4–0.8% FOS) (39). The distinction between these and the current findings may be because of FOS additive levels, the fish species used, and the experimental conditions.

Lysozyme is a hydrolase enzyme produced by leucocytes, predominantly neutrophils and macrophages. It is an essential parameter in the innate immune defense of both invertebrates and vertebrates. In fish, this enzyme can be found in the mucus, the lymphoid tissues, plasma and other fluid components of a body (40). In this study, lysozyme activity significantly increased in Nile tilapia supplemented with 5 g/kg of FOS, 3 × 10^9^ CFU/g *B. subtilis*, and 5 × 10^9^ CFU/g *B. subtilis*. The dose, feeding time, composition, and source need to be considered for prebiotic and probiotic feed addition because responses may vary depending on species, size, age, and physiological status. Previous studies have reported that prebiotics and probiotics, either singly or in combination, can stimulate an increase in lysozyme levels or stimulate macrophages, which are the primary producers of lysozyme in fish. Caspian roach fry fed 2% and 3% FOS for 7 weeks had significantly greater lysozyme activity than the 1% FOS and the control group (41). The effects of FOS on various innate immune responses, including phagocytosis, lysozyme activity, and the complement system activity in *Sparus aurata* and *Dicentrarchus labrax*, were reported (30). In addition, the dietary supplementation with 1 × 10^4^ and 1 × 10^6^ CFU/g *B. amyloliquefaciens* spores significantly improved lysozyme activity in Nile tilapia after 15 and 30 days of feeding (42). Thus, prebiotic and probiotic supplementation at an appropriate concentration possibly enhanced lysozyme activity in fish.

Probiotics could enhance phagocytic activity in many aquatic animals. The respiratory burst activity of Nile tilapia treated with *Bacillus* sp. KUAQ and *Bacillus* sp. KUAQ2 containing 3 × 10 ^8^ CFU/g of feed (3) and Nile tilapia fed with *B. subtilis* at a dose of 1 × 10 ^7^ CFU/g of feed was significantly higher than in those of the control. In addition to probiotics in aquaculture, prebiotics FOS, MOS, β-glucan, and GOS are also used as feed additives to stimulate immune responses. According to previous reports, Caspian roach (*Rutilus rutilus*) fed 2% and 3% of FOS and Common carp (*Cyprinus carpio*) fed with 2% FOS (43) showed significantly increased levels of respiratory burst activity compared to a control group (*P* < 0.05). The mechanism of immune responses starts when bacterial cell wall components such as lipopolysaccharides or peptidoglycans have adhered to the binding proteins in a host, and the binding complexes are then recognized by recognition proteins. After these reaction processes, the immune function, such as phagocytosis, can be activated (44). In addition, *Bacillus* sp. can synthesize various vitamins, which may affect the leucocytes and enhance lysozyme and respiratory burst activity (45).

The complement system is a major component of innate humoral immunity modulation and has a vital role in host homeostasis, inflammation, antibody opsonization, and in the defense against pathogens. It consists of three activation pathways: the classical pathway, lectin pathway, and alternative pathway (46). The complement component 3 (C3) gene is responsible for producing a protein that plays an essential role in immune system regulation and pathology (47, 48). Probiotic *B. subtilis* and FOS could stimulate complement C3 gene expression levels in livers and spleens (49), which are the main organs for C3 synthesis. In this study, the enhancement of C3 expression in livers was noticed in fish fed with 5 g FOS/kg feed and those fed with *Bacillus* supplementary diets. This result agrees with previous reports on teleost C3, which pointed out that the liver and spleen are generally considered the prime organs involved in C3 synthesis (50). C3 levels in groupers (*Epinephelus coioides)* fed with *Bacillus* spp. were significantly higher than that of the control after 30 days of feeding (51). In addition, after 3 weeks of *B. subtilis* supplementary feeding, complement activity in Gilthead seabream (*S. aurata* L.) improved compared with controls (52). The expression of C3 was significantly up-regulated in the liver and spleen after challenging the southern catfish (*Silurus meridionalis*) with *A. hydrophila* (50). Greater C3 levels can help grass carp better cope with secondary infections of *A. hydrophila*, allowing them to survive. Prebiotic and probiotic metabolites could stimulate C3 complement after being directly activated by bacterial lipopolysaccharide and subsequently this resulted in the direct killing of pathogens by lysis (53).

IL-1β and TNF-α are cytokines required for activating the innate immune response, mediating the recruitment, activation, and adherence of circulating phagocytic cells, responsible for inflammation activity, neutrophil activation, and microbial killing of both gram-positive and negative bacteria (54). The results of this study showed that expression of IL-1β and TNF-α was affected by the application of 5 g FOS/kg feed and *Bacillus* additive diets significantly. *IFN-*γ is one of antiviral cytokines and functions as the primary activator of macrophages. The expression of *IL-1, IFN-*γ and *TNF-*α genes in the head kidney of *C. auratus* fed with *B. velezensis* at a density of 10^9^ CFU/g was shown to be increased (55). This was also true for Japanese seabass fed with *B. pumillus* SE5 fermented soybean (56), and for Nile tilapia fed *A. oryzae* at 1 × 10^6^ or 1 × 10^8^ CFU/g (31).

Administering FOS and *B. subtilis* enhanced the expression levels of liver *hsp70* gene in fish, potentially strengthening their tolerance to environmental stressors such as heat, disease, parasitic infection, and chemical exposure. The *hsp70* gene expression level was higher for fish fed with FOS 5 g/Kg feed and for all *B. subtilis* addition groups (*P* < 0.05) in this study. The results are similar to those of previously reported studies, Nile tilapia fed with *B. subtilis* and *B. licheniformis*, mixed in a ratio of 1:1 w/w at 10 g/kg showed the greater expression of the *hsp70* gene in the head-kidney (57). In addition, the liver *hsp70* expression of blunt snout bream fed 0.4 % FOS was significantly enhanced under high heat stress, ambient temperature +8°C (58). High levels of *hsp70* possibly indicated high levels of protein damage and increased tolerance to subsequent stress and others (59). *hsp70* is an effective tool for helping in the survival rates of cells through stress protection, cures, and environmental pressure relief (60).

*S. agalactiae* is considered a critical bacterial disease causing high mortality rates and economic losses in tilapia. The challenge test is used as an ultimate assay to assess the fish immune response. Although the highest survival rate was noticed in tilapia fed with 3 × 10^9^ CFU/g *B. subtilis* group; however, there were no significant differences. Similarly, fish were fed with probiotics, this did not increase the survival rate of tilapia challenged with *S. agalactiae* (3). In addition, the combined feeding with *B. subtilis* strains SB3086 and SB3615 did not result in any significant difference in reducing mortality due to *S. iniae* infection in juvenile Nile tilapia (61). FOS and *B. licheniformis*, used as prebiotic and probiotic, did not significantly influence (*P* > 0.05) the survival rate of triangular bream after a *A. hydrophila* challenge (59). On the other hand, 10 g/kg of a mix of *B. subtilis and B. licheniformis* application results in significantly greater survival of tilapia against *Streptococcus agalactiae* (57). The differences in pathogen prevention may be due to FOS additive levels, purity, sources, the fish species used, pathogen virulence, and the experimental conditions. Moreover, a non-significant increase in the protection level of FOS and *B. subtilis* supplemented groups against *S. agalactiae* although immunity was improved. The possible explanation could be all immune-related gene expression applied in this study was the first line of non-specific defense, possibly this expression or defense mechanism was not strong enough to protect the fish from deadly pathogens or maybe this pathogen was very virulent.

## 5. Conclusion

In conclusion, a feed containing FOS and *B. subtilis* showed no significant effects on overall growth performances in Tilapia. However, significant effects were observed on the expression of immune-related genes, including Complement C3, *IL-1*β, *TNF-*α, *IFN-*γ, and *hsp70* genes; it may also increase their resistance to *S. agalactiae*. Thus, further investigation of other prebiotics or herbs in combination with *B. subtilis* is encouraged at molecular levels and screening for beneficial metabolites that may stimulate digestive enzymes, growth, and health benefits in tilapia.

## Data availability statement

The raw data supporting the conclusions of this article will be made available by the authors, without undue reservation.

## Ethics statement

The animal study was reviewed and approved by Maejo University.

## Author contributions

AP was responsible for investigation, writing the original draft, and visualization. CC was in charge of conceptualization, validation, data curation, writing—review and editing, visualization, supervision, project administration, and funding acquisition. All authors were involved in methodology, investigation, and reviewed the manuscript. All authors contributed to the article and approved the submitted version.

## References

[1] Chen SW Liu CH Hu SY. Dietary administration of probiotic *Paenibacillus ehimensis* Npust1 with bacteriocin-like activity improves growth performance and immunity against *Aeromonas hydrophila* and *Streptococcus iniae* in Nile tilapia (*Oreochromis niloticus*). Fish Shellfish Immunol. (2019) 84:695–703. 10.1016/j.fsi.2018.10.059 30368025

[2] Prabu E Rajagopalsamy C Ahilan B Jeevagan IJMA Tilapia–an excellent candidate species for world aquaculture: a review. Annu Res Rev Biol. (2019) 31:1–14. 10.9734/arrb/2019/v31i330052

[3] Sookchaiyaporn N Srisapoome P Unajak S Areechon N. Efficacy of *Bacillus* spp. isolated from Nile tilapia *Oreochromis niloticus* Linn. on its growth and immunity, and control of pathogenic bacteria. Fish Sci. (2020) 86:353–65. 10.1007/s12562-019-01394-0

[4] Kraemer SA Ramachandran A Perron GG. Antibiotic Pollution in the environment: from microbial ecology to public policy. Microorganisms. (2019) 7:180. 10.3390/microorganisms7060180 31234491 PMC6616856

[5] Lindsey APJ Murugan S Renitta RE. Microbial disease management in agriculture: current status and future prospects. Biocatal Agric Biotechnol. (2020) 23:101468. 10.1016/j.bcab.2019.101468

[6] Defoirdt T Sorgeloos P Bossier P. Alternatives to antibiotics for the control of bacterial disease in aquaculture. Curr Opin Microbiol. (2011) 14:251–8. 10.1016/j.mib.2011.03.004 21489864

[7] Mohan K Ravichandran S Muralisankar T Uthayakumar V Chandirasekar R Seedevi P. Potential uses of fungal polysaccharides as immunostimulants in fish and shrimp aquaculture: a review. Aquac. (2019) 500:250–63. 10.1016/j.aquaculture.2018.10.023 30599257

[8] Van Doan H Hoseinifar SH Esteban MÁ Dadar M Thu TTN. Mushrooms, seaweed, and their derivatives as functional feed additives for aquaculture: an updated view. Stud Nat Prod Chem. (2019) 62:41–90. 10.1016/B978-0-444-64185-4.00002-2

[9] Pandiyan P Balaraman D Thirunavukkarasu R George EGJ Subaramaniyan K Manikkam S. Probiotics in aquaculture. Drug Discov Today. (2013) 5:55–9. 10.1016/j.dit.2013.03.003

[10] Dawood MA Abo-Al-Ela HG Hasan MT. Modulation of transcriptomic profile in aquatic animals: probiotics, prebiotics and synbiotics scenarios. Fish Shellfish Immunol. (2020) 97:268–82. 10.1016/j.fsi.2019.12.054 31863903

[11] Gibson GR Probert HM Van Loo J Rastall RA Roberfroid MB. Dietary modulation of the human colonic microbiota: updating the concept of prebiotics. Nutr Res Rev. (2004) 17:259–75. 10.1079/NRR200479 19079930

[12] Grisdale-Helland B Helland SJ Gatlin III DM. The effects of dietary supplementation with mannanoligosaccharide, fructooligosaccharide or galactooligosaccharide on the growth and feed utilization of atlantic salmon (*Salmo salar*). Aquaculture. (2008) 283:163–7. 10.1016/j.aquaculture.2008.07.012

[13] Ringø E Olsen R Gifstad T Dalmo R Amlund H Hemre GI. Prebiotics in aquaculture: a review. Aquac Nut. (2010) 16:117–36. 10.1111/j.1365-2095.2009.00731.x

[14] El-latif A Ashraf M El-Gawad A Emam M. Effect of dietary fructooligosaccharide supplementation on feed utilization and growth performance of Nile tilapia (*Oreochromis niloticus*) Fingerlings. Egy J Aquac. (2015) 5:1–16. 10.21608/eja.2019.46730

[15] Abd El-Gawad EA Abd El-latif AM Amin AA Abd-El-Azem M. E effect of dietary fructooligosaccharide on bacterial infection, oxidative stress and histopathological alterations in Nile tilapia (*Oreochromis niloticus*). Glob Vet. (2015) 15:339–50. 10.5829/idosi.gv.2015.15.04.10122

[16] Abd El-Gawad EA Abd El-latif AM Shourbela RM. Enhancement of antioxidant activity, non-specific immunity and growth performance of Nile tilapia, *Oreochromis niloticus* by dietary fructooligosaccharide. J Aquac Res Dev. (2016) 7:1–7. 10.4172/2155-9546.1000427

[17] Soleimani N Hoseinifar SH Merrifield DL Barati M Abadi ZH. Dietary supplementation of fructooligosaccharide (FOS) improves the innate immune response, stress resistance, digestive enzyme activities and growth performance of Caspian Roach (*Rutilus rutilus*) fry. Fish Shellfish Immunol. (2012) 32:316–21. 10.1016/j.fsi.2011.11.023 22142706

[18] de Lima Paz A da Silva JM da Silva KMM Val AL. Protective effects of the fructooligosaccharide on the growth performance, hematology, immunology indicators and survival of Tambaqui (*Colossoma macropomum*, Characiformes: Serrasalmidae) infected by *Aeromonas hydrophila*. Aquac Rep. (2019) 15:100222. 10.1016/j.aqrep.2019.100222

[19] Akrami R Iri Y Rostami HK Mansour MR. Effect of dietary supplementation of fructooligosaccharide (FOS) on growth performance, survival, lactobacillus bacterial population and hemato-immunological parameters of Stellate Sturgeon (*Acipenser stellatus*) juvenile. Fish Shellfish Immunol. (2013) 35:1235–9. 10.1016/j.fsi.2013.07.039 23973846

[20] Kuebutornye FK Abarike ED Lu Y. A review on the application of *Bacillus* as probiotics in aquaculture. Fish Shellfish Immunol. (2019) 87:820–8. 10.1016/j.fsi.2019.02.010 30779995

[21] Elshaghabee FM Rokana N Gulhane RD Sharma C Panwar H. *Bacillus* as potential probiotics: status, concerns, and future perspectives. Front Microbiol. (2017) 8:1490. 10.3389/fmicb.2017.01490 28848511 PMC5554123

[22] Zaineldin AI Hegazi S Koshio S Ishikawa M Bakr A El-Keredy AM. *Bacillus subtilis* as probiotic candidate for red sea bream: growth performance, oxidative status, and immune response traits. Fish Shellfish Immunol. (2018) 79:303–12. 10.1016/j.fsi.2018.05.035 29792927

[23] Apún-Molina JP Santamaría-Miranda A Luna-González A Martínez-Díaz SF Rojas-Contreras M. Effect of potential probiotic bacteria on growth and survival of Tilapia *Oreochromis niloticus* L., cultured in the laboratory under high density and suboptimum temperature. Aquac Res. (2009) 40:887–94. 10.1111/j.1365-2109.2009.02172.x

[24] He S Zhang Y Xu L Yang Y Marubashi T Zhou Z. Effects of dietary bacillus subtilis C-3102 on the production, intestinal cytokine expression and autochthonous bacteria of hybrid Tilapia *Oreochromis niloticus*♀ × *Oreochromis aureus*♂. Aquaculture. (2013) 412:125–30. 10.1016/j.aquaculture.2013.06.028

[25] Telli GS Ranzani-Paiva MJT de Carla Dias D Sussel FR Ishikawa CM Tachibana L. Dietary administration of *Bacillus subtilis* on hematology and non-specific immunity of Nile tilapia *Oreochromis niloticus* raised at different stocking densities. Fish Shellfish Immunol. (2014) 39:305–11. 10.1016/j.fsi.2014.05.025 24878743

[26] Ng WK Kim YC Romano N Koh C-B Yang S-Y. Effects of dietary probiotics on the growth and feeding efficiency of red hybrid Tilapia, *Oreochromis* sp., and subsequent resistance to *Streptococcus agalactiae. J Appl Aquac*. (2014) 26:22–31. 10.1080/10454438.2013.874961

[27] Iwashita MKP Nakandakare IB Terhune JS Wood T Ranzani-Paiva MJT. Dietary Supplementation with *Bacillus subtilis, Saccharomyces cerevisiae* and *Aspergillus oryzae* enhance immunity and disease resistance against *Aeromonas hydrophila* and *Streptococcus iniae* infection in juvenile tilapia *Oreochromis niloticus*. Fish Shellfish Immunol. (2015) 43:60–6. 10.1016/j.fsi.2014.12.008 25530581

[28] Secombes C. Isolation of salmonid macrophages and analysis of their killing activity. Techn Fish Immunol. (1990):137–54.

[29] Livak KJ Schmittgen TD. Analysis of relative gene expression data using real-time quantitative Pcr and the 2– Δδct method. Methods. (2001) 25:402–8. 10.1006/meth.2001.1262 11846609

[30] Carbone D Faggio C. Importance of prebiotics in aquaculture as immunostimulants. effects on immune system of *Sparus aurata* and *Dicentrarchus labrax*. Fish Shellfish Immunol. (2016) 54:172–8. 10.1016/j.fsi.2016.04.011 27074444

[31] Dawood MA Eweedah NM Moustafa EM Farahat EM. Probiotic effects of *Aspergillus oryzae* on the oxidative status, heat shock protein, and immune related gene expression of Nile tilapia (*Oreochromis niloticus*) under hypoxia challenge. Aquaculture. (2020) 520:734669. 10.1016/j.aquaculture.2019.734669

[32] Ringø E. Probiotics in shellfish aquaculture. Aquac Fish. (2020) 5:1–27. 10.1016/j.aaf.2019.12.001

[33] Ringø E Van Doan H Lee SH Soltani M Hoseinifar SH Harikrishnan R. Probiotics, lactic acid bacteria and bacilli: interesting supplementation for aquaculture. J Appl Microbiol. (2020) 129:116–36. 10.1111/jam.14628 32141152

[34] Wang Y Wang Q Xing K Jiang P Wang J. Dietary cinnamaldehyde and *Bacillus subtilis* improve growth performance, digestive enzyme activity, and antioxidant capability and shape intestinal microbiota in tongue sole, *Cynoglossus semilaevis*. Aquaculture. (2021) 531:735798. 10.1016/j.aquaculture.2020.735798

[35] Büyükdeveci ME Cengizler I Balcázar JL Demirkale I. Effects of two host-associated probiotics *Bacillus mojavensis* B191 and *Bacillus subtilis* Mrs11 on growth performance, intestinal morphology, expression of immune-related genes and disease resistance of Nile tilapia (*Oreochromis niloticus*) against *Streptococcus iniae*. Dev Comp Immunol. (2023) 138:104553. 10.1016/j.dci.2022.104553 36122732

[36] El-Son MA Elshopakey GE Rezk S Eldessouki EA Elbahnaswy S. Dietary mixed *Bacillus* strains promoted the growth indices, enzymatic profile, intestinal immunity, and liver and intestinal histomorphology of Nile tilapia, *Oreochromis niloticus*. Aquac Rep. (2022) 27:101385. 10.1016/j.aqrep.2022.101385

[37] Liu H Wang S Cai Y Guo X Cao Z Zhang Y. Dietary administration of *Bacillus subtilis* Hainup40 enhances growth, digestive enzyme activities, innate immune responses and disease resistance of Tilapia, *Oreochromis niloticus*. Fish Shellfish Immunol. (2017) 60:326–33. 10.1016/j.fsi.2016.12.003 27919757

[38] Ai Q Xu H Mai K Xu W Wang J Zhang W. Effects of dietary supplementation of *bacillus subtilis* and fructooligosaccharide on growth performance, survival, non-specific immune response and disease resistance of juvenile large yellow croaker, *Larimichthys crocea*. Aquaculture. (2011) 317:155–61. 10.1016/j.aquaculture.2011.04.036

[39] Zhang C-N Li X-F Jiang G-Z Zhang D-D Tian H-Y Li J-Y. Effects of dietary fructooligosaccharide levels and feeding modes on growth, immune responses, antioxidant capability and disease resistance of Blunt Snout Bream (*Megalobrama amblycephala*). Fish Shellfish Immunol. (2014) 41:560–9. 10.1016/j.fsi.2014.10.005 25451000

[40] Magnadóttir B. Innate immunity of fish (overview). Fish Shellfish Immunol. (2006) 20:137–51. 10.1016/j.fsi.2004.09.006 15950491

[41] Akhter N Wu B Memon AM Mohsin M. Probiotics and prebiotics associated with aquaculture: a review. Fish Shellfish Immunol. (2015) 45:733–41. 10.1016/j.fsi.2015.05.038 26044743

[42] Selim KM Reda RM. Improvement of immunity and disease resistance in the Nile tilapia, *Oreochromis niloticus*, by dietary supplementation with *Bacillus amyloliquefaciens*. Fish Shellfish Immunol. (2015) 44:496–503. 10.1016/j.fsi.2015.03.004 25783002

[43] Hoseinifar SH Ahmadi A Raeisi M Hoseini SM Khalili M Behnampour N. Comparative study on immunomodulatory and growth enhancing effects of three prebiotics (Galactooligosac charide, Fructooligosaccharide and Inulin) in common carp (*Cyprinus carpio*). Aquac Res. (2017) 48:3298–307. 10.1111/are.13156

[44] Vargas-Albores F Yepiz-Plascencia G. Beta glucan binding protein and its role in shrimp immune response. Aquaculture. (2000) 191:13–21. 10.1016/S0044-8486(00)00416-6

[45] Hoseinifar SH Sun Y-Z Wang A Zhou Z. Probiotics as means of diseases control in aquaculture, a review of current knowledge and future perspectives. Front Microbiol. (2018) 9:2429. 10.3389/fmicb.2018.02429 30369918 PMC6194580

[46] Noris M Remuzzi G editors. Overview of complement activation and regulation. Semin Nephrol. (2013) 33:479–92. 10.1016/j.semnephrol.2013.08.001 24161035 PMC3820029

[47] Ricklin D Reis ES Mastellos DC Gros P Lambris JD. Complement component C3–the “swiss army knife” of innate immunity and host defense. Immunol Rev. (2016) 274:33–58. 10.1111/imr.12500 27782325 PMC5427221

[48] Meng X Shen Y Wang S Xu X Dang Y Zhang M. Complement component 3 (C3): an important role in grass carp (*Ctenopharyngodon idella*) experimentally exposed to *Aeromonas hydrophila*. Fish Shellfish Immunol. (2019) 88:189–97. 10.1016/j.fsi.2019.02.061 30826411

[49] Zhang Q Yu H Tong T Tong W Dong L Xu M. Dietary supplementation of *Bacillus subtilis* and fructooligosaccharide enhance the growth, non-specific immunity of juvenile Ovate Pompano, *Trachinotus ovatus* and its disease resistance against *Vibrio vulnificus*. Fish Shellfish Immunol. (2014) 38:7–14. 10.1016/j.fsi.2014.02.008 24614017

[50] Fu YW Zhu CK Zhang QZ. Molecular characterization and expression analysis of complement component C3 in Southern Catfish (*Silurus meridionalis*) and a whole mount *in situ* hybridization study on its ontogeny. Fish Shellfish Immunol. (2019) 84:865–75. 10.1016/j.fsi.2018.10.083 30389643

[51] Sun YZ Yang HL Ma RL Lin WY. Probiotic applications of two dominant gut *Bacillus* strains with antagonistic activity improved the growth performance and immune responses of Grouper *Epinephelus coioides*. Fish Shellfish Immunol. (2010) 29:803–9. 10.1016/j.fsi.2010.07.018 20637875

[52] Salinas I Abelli L Bertoni F Picchietti S Roque A Furones D. Monospecies and multispecies probiotic formulations produce different systemic and local immunostimulatory effects in the Gilthead Seabream (*Sparus aurata* L.). Fish Shellfish Immunol. (2008) 25:114–23. 10.1016/j.fsi.2008.03.011 18442923

[53] Nayak SK. Probiotics and immunity: a fish perspective. Fish Shellfish Immunol. (2010) 29:2–14. 10.1016/j.fsi.2010.02.017 20219683

[54] Tort L Balasch J Mackenzie S. Fish immune system. A crossroads between innate and adaptive responses. Inmunologia. (2003) 22:277–86.32536909

[55] Yi Y Zhang Z Zhao F Liu H Yu L Zha J. Probiotic potential of *Bacillus velezensis* Jw: antimicrobial activity against fish pathogenic bacteria and immune enhancement effects on *Carassius auratus*. Fish Shellfish Immunol. (2018) 78:322–30. 10.1016/j.fsi.2018.04.055 29702236

[56] Rahimnejad S Lu K Wang L Song K Mai K Davis DA. Replacement of fish meal with *Bacillus pumillus* SE5 and *Pseudozyma aphidis* ZR1 fermented soybean meal in diets for Japanese Seabass (*Lateolabrax japonicus*). Fish Shellfish Immunol. (2019) 84:987–97. 10.1016/j.fsi.2018.11.009 30403972

[57] Abarike ED Cai J Lu Y Yu H Chen L Jian J. Effects of a commercial probiotic Bs containing *Bacillus subtilis* and *Bacillus licheniformis* on growth, immune response and disease resistance in Nile tilapia*, Oreochromis niloticus*. Fish Shellfish Immunol. (2018) 82:229–38. 10.1016/j.fsi.2018.08.037 30125705

[58] Zhang CN Li XF Tian HY Zhang DD Jiang GZ Lu KL. Effects of fructooligosaccharide on immune response, antioxidant capability and Hsp70 and Hsp90 expressions of Blunt Snout Bream (*Megalobrama amblycephala*) under high ammonia stress. Fish Physiol Biochem. (2015) 41:203–17. 10.1007/s10695-014-0017-6 25432579

[59] Zhang CN Li XF Xu WN Jiang GZ Lu KL Wang LN. Combined effects of dietary fructooligosaccharide and *Bacillus licheniformis* on innate immunity, antioxidant capability and disease resistance of Triangular Bream (*Megalobrama terminalis*). Fish Shellfish Immunol. (2013) 35:1380–6. 10.1016/j.fsi.2013.07.047 23932988

[60] Lindquist S Craig EA. The heat-shock proteins. Annu Rev Genet. (1988) 22:631–77. 10.1146/annurev.ge.22.120188.003215 2853609

[61] Addo S Carrias AA Williams MA Liles MR Terhune JS Davis DA. Effects of *Bacillus subtilis* strains on growth, immune parameters, and *Streptococcus iniae* susceptibility in Nile tilapia, *Oreochromis niloticus*. J World Aquac Soc. (2017) 48:257–67. 10.1111/jwas.12380

[62] Phumyu N Boonanuntanasarn S Jangprai A Yoshizaki G Na-Nakorn U. Pubertal effects of 17α-methyltestosterone on Gh–Igf-related genes of the hypothalamic–pituitary–liver–gonadal axis and other biological parameters in male, female and sex-reversed Nile tilapia. Gen Comp Endocrinol. (2012) 177:278–92. 10.1016/j.ygcen.2012.03.008 22481004

[63] Kayansamruaj P Dong HT Pirarat N Nilubol D Rodkhum C. Efficacy of α-enolase-based DNA vaccine against pathogenic *Streptococcus iniae* in Nile tilapia (*Oreochromis niloticus*). Aquaculture. (2017) 468:102–6. 10.1016/j.aquaculture.2016.10.001

[64] Chen Y-B Hu J Lyu Q-J Liu L-J Wen L-F Yang X-K. The effects of natucin C-natucin p mixture on blood biochemical parameters, antioxidant activity and non-specific immune responses in Tilapia (*Oreochromis niloticus*). Fish Shellfish Immunol. (2016) 55:367–73. 10.1016/j.fsi.2016.06.016 27298271

